# What women think: hypothetical notions of screening for intimate partner violence in Kenyan hospital settings

**DOI:** 10.1186/1753-6561-9-S4-A6

**Published:** 2015-07-07

**Authors:** Chi-Chi Undie, M Catherine Maternowska, Margaret Mak'anyengo, Ian Askew

**Affiliations:** 1 Population Council, P.O. Box 17643-00500, Nairobi, Kenya; 2 Child Protection, Research and Evaluation, UNICEF, Office of Research-Innocenti, 50122 Florence, Italy; 3 Bixby Center for Global Reproductive Health, University of California, San Francisco, CA, USA; 4 Gender-Based Violence Recovery Centre, Kenyatta National Hospital, P. O. Box 20723-00202, Nairobi, Kenya

## Background

Although screening for intimate partner violence (IPV) is current health policy in northern countries [1], developing country contexts such as Kenya have yet to adopt this practice. There are good reasons for this hesitation, given that strong health systems should ideally be in place prior to the introduction of such screening. However, the current emergence of one-stop centers for gender-based violence in African countries now provides an opportunity to explore the utility of screening in a region where IPV has become an increasingly prevalent public health concern [2]. It also creates room for considering the opinions of women - a population for whom IPV is the most common form of violence experienced globally [3] - in order to inform models of care for IPV survivors in African contexts. This study examined the hypothetical utility of IPV screening (coupled with referral for IPV care) from the perspective of women seeking services at Kenyatta National Hospital in Nairobi, Kenya - the oldest and largest public referral, teaching, and research hospital in the East African region.

## Materials and methods

The study involved 68 semi-structured, in-depth interviews with purposively-selected women aged 18 years and above, who sought services at one of four clinics at Kenyatta National Hospital during the data collection period of April to June, 2011: the antenatal care clinic, the HIV comprehensive care clinic, the Gender-Based Violence Recovery Center (a one-stop center for gender-based violence), and the Youth Center (a youth-friendly reproductive health and HIV clinic). Data were collected by two female interviewers who were not involved in the clinical management of the respondents. These data were generated in response to hypothetical questions posed to women to assess the extent to which IPV screening would be acceptable to, and useful for, them. The in-depth interviews were hand-recorded verbatim and typed up in MS Word. Data were analyzed using content analysis techniques primarily, with codes developed and configured along lines of topical inquiry. Additional codes were developed based on emerging themes that seemed important for determining the utility of IPV screening. Dominant themes depicting women's main thoughts about IPV screening are presented here.

## Results: what women think

Qualitative analysis suggested that women's opinions about hypothetical IPV screening protocols in this Kenyan context are shaped by their concerns about confidentiality; the need to raise awareness around IPV and available care; gaps in the health care system that translate to unmet needs of survivors; and the need for clear-cut responses to IPV that do not promise more than can be realistically offered. While there is some overlap between these themes, each is presented separately, and verbatim quotes from respondents are labelled according to the location at which the respondent sought care.

### Provider distance could help facilitate screening

The women in this study perceived that the involvement of health providers (particularly - but not solely - doctors) in the screening and referral process would serve to greatly enhance the chances of survivors complying with referrals for IPV care. Providers in hospital settings were seen by respondents as highly respected due to their specialized training, and able to maintain professional distance and confidentiality. Consequently, a provider's recommendation that a survivor access IPV services was viewed as particularly persuasive:

You can use doctors to convince [survivors] to come [for IPV services]. Patients really respect doctors and they will listen to them (Client, Gender-Based Violence Recovery Center).

[A survivor] can tell the doctor or nurse about her IPV experience and [they] will not gossip about her because they are strangers (Client, Antenatal Care Clinic).

### Screening would enhance awareness of IPV

Another major feature of respondents' narratives had to do with women regarding the screening process as educational: whether one was experiencing violence or not, screening would inadvertently alert those screened to the health implications of IPV, while enhancing awareness of where assistance and services can be obtained. Respondents strongly perceived that women (survivors and non-survivors alike) would be likely to help others by sharing information received during screening with others.

Even if I'm not experiencing violence right now, those services will help other women, and some of them could be my friends. And if you know about these services, you can tell other women who may not know about [them] (Client, Antenatal Care Clinic).

You never know about life. I may go through it in [the] future and I'll be able to know where to go for help. I can also tell my friends about it and they will be able to get help before something serious happens to them (Client, Youth Center).

### The screening process would be cathartic

Overwhelmingly, the perceived cathartic effect expected from a successful IPV screening and referral process was highlighted by respondents as a factor that would make screening worthwhile. This hypothetical process was viewed by participants as a rare opportunity for survivors to speak to someone about a taboo issue. This prospect was strongly viewed as sufficient to motivate any survivor to hold a positive view of screening protocols.

I know many women will not refuse [to be screened]... because most women experience [IPV], but they may have never had an opportunity of someone asking them about it in a hospital set-up (Client, Antenatal Care Clinic).

In most cases, women are never given a chance to talk. Many are harassed sexually, but society has no avenues to make them talk. This will be a chance for them to do so (Client, Youth Center).

[W]omen have many problems and they are just waiting for an opportunity for someone they can share with so as to release the stress they have been carrying (Client, HIV Comprehensive Care Center).

### There is already hidden demand for IPV services

The sheer desire for assistance as a catalyst toward IPV services uptake emerged as a recurrent theme among respondents. Here, women highlighted the fact that IPV screening holds potential for capturing those survivors that represent 'low hanging fruit,' in the sense that they are ripe for intervention and require no coaxing beyond the provision of information about the availability of services:

If a woman is suffering because of her husband and she wants to be helped, she will go [for IPV care] because she wants somebody to help her (Client, Antenatal Care Clinic).

If she wants to be helped, if she wants a breather in her life from what she has been going through, then she will go (Client, Youth Center).

### Being clear about any post-screening benefits would be important

Some women conceptualized help for IPV in the form of non-medical responses, presuming that presenting for IPV care would accrue particular benefits to survivors. These imagined benefits ranged from rapid legal assistance, to marriage counseling, and financial assistance:

I can also get help because of what I'm going through ... in my marriage. ... Maybe you are setting up small businesses for women who are being abused and I can be helped through that (Client, HIV Comprehensive Care Center).

[Some] will think that women will be given money for food or school fees, or get benefits like ... money for business (Client, Antenatal Care Clinic).

The need for clarity around what a survivor can expect to gain from following through with IPV referrals was overwhelmingly emphasized by respondents in order to avoid misconceptions and to underscore IPV as a health issue for which medical attention should be sought.

## Conclusions

The perspectives generated by this study - although hypothetical - suggest that various facilitators to potential IPV screening processes in Kenya exist: Women are highly amenable to being screened by doctors or nurses (who, incidentally, would be the most likely candidates to take on screening roles in this setting). Women appreciate the role that IPV screening would inadvertently play in creating awareness of this phenomenon as a health issue, and of available services for addressing it. The women interviewed in this study were convinced that there was latent demand among their peers for IPV services, and that accessing such services after identification through screening would be cathartic for survivors. Lastly, women strongly emphasized the need to clarify the content of services available to survivors early on in the screening process, in order to avoid misconceptions and unrealistic expectations.

The perspectives of Kenyan women on conducting IPV screening in Kenyan health care settings support the findings of studies in non-African contexts that highlight the critical importance of women's voices in planning for female-centered screening interventions. The voices shared in this paper point to the potential value of IPV screening in health care settings in the sub-Saharan Africa region, while also highlighting the need for a cautionary approach in designing such screening interventions.

Findings from this and other studies centering on the acceptability and feasibility of IPV screening in East African countries [4-7] were presented as part of a special panel at the 6^th ^Best Practices Forum in Health of the East, Central and Southern Africa (ECSA) Health Community in August 2012. Deliberations over these findings led to the passage of a resolution (in December 2012) by Health Ministers from the ECSA region, calling for the integration of gender-based violence screening into sexual and reproductive health and HIV and AIDS services in the region, coupled with support for Member States to effect such integration (ECSA/HMC56/R2, Number 5 [ECSA Secretariat] and Number 7 [ECSA Member States]).

Since that time, findings from this exploratory study have been used to inform the development of an IPV screening intervention at Kenyatta National Hospital, which was proven to be feasible and effective [8]. Further funding was obtained to adapt this screening intervention for humanitarian settings in Uganda, and to the needs of child survivors of sexual violence in Kenya. Both efforts (in Uganda and Kenya) involve assessments to determine feasibility and effectiveness of the screening intervention in new settings.
